# When sport becomes social: how exercise and live spectatorship are linked to happiness through neighborhood connections

**DOI:** 10.3389/fpubh.2026.1937960

**Published:** 2026-09-09

**Authors:** Huiying Song, Zhengbing Guo

**Affiliations:** 1 Xi'an Siyuan University, Xi'an, China; 2 School of Humanities and Social Science, Xi’an Jiaotong University, Xi'an, China

**Keywords:** happiness, live sports spectatorship, neighborhood social capital, sport involvement, structured exercise participation

## Abstract

**Introduction:**

Sport is often understood as a health behavior associated with happiness through physical activity and psychological regulation. However, less is known about the social pathways connecting different forms of sport involvement with happiness. This study examined the associations of structured exercise participation (SEP) and live sports spectatorship (LSS) with happiness and investigated the mediating role of neighborhood social capital (NSC).

**Methods:**

Data were drawn from 5,451 respondents in the 2023 Chinese General Social Survey. OLS regression was used as the main analytical approach, ordered logistic regression was conducted as a robustness check, and bootstrap mediation analysis was used to estimate the indirect, direct, and total associations of SEP and LSS with happiness through NSC.

**Results:**

Both SEP and LSS were positively associated with happiness after adjustment for demographic, socioeconomic, health-related, and residential characteristics. The ordered logistic models produced substantively consistent results. Bootstrap analysis showed significant positive indirect associations through NSC for both SEP and LSS. For SEP, the direct association remained statistically significant after NSC was included. For LSS, the direct association was not statistically significant, whereas the indirect and total associations remained significant.

**Discussion:**

The findings suggest that sport involvement is associated with happiness not only through active physical participation or spectator-based leisure, but also through neighborhood social connections and everyday social embeddedness. SEP appears to be linked to happiness through both direct and socially mediated pathways, whereas the association of LSS with happiness appears to operate primarily through NSC. Given the cross-sectional design, these findings should be interpreted as associations rather than causal effects.

## 1 Introduction

In contemporary China, improving residents’ happiness has become an increasingly important issue in public health, social governance, and everyday life. As social mobility accelerates and digital life expands, individuals are increasingly exposed to fragmented routines, weakened local ties, and the risk of social isolation. Social isolation and loneliness have been shown to undermine happiness, especially when everyday social support is insufficient (1). Neighborhood-based social resources can help sustain life satisfaction by embedding individuals in local networks of interaction and support (2, 3). From this perspective, happiness should not be understood simply as the outcome of physical health or individual psychological adjustment. It is also shaped by everyday interaction, social embeddedness, and the quality of local life. Sport, therefore, should not be reduced to calories burned on a treadmill or to a purely biological mechanism of health improvement. Physical exercise is related to life satisfaction among Chinese urban residents, but this relationship also reflects broader lifestyle and social conditions (4). Sport can also operate as a social practice embedded in shared spaces, repeated encounters, and collective routines. Participation in sport-related events may generate social capital by creating opportunities for interaction, recognition, and shared experience (5). To understand how sport contributes to happiness, it is therefore necessary to place sport back into the social field in which it occurs.

Yet the social meaning of sport involvement is more diverse than the conventional focus on physical exercise suggests. Modern residents engage with sport through at least two distinct modes. One is structured exercise participation (SEP), through which individuals take part in gym-based or venue-based exercise and reshape their bodily routines, spatial participation, and everyday order. The other is live sports spectatorship (LSS), through which individuals enter stadiums or event spaces as spectators and share collective attention, emotional excitement, and symbolic identification with others. These two modes represent different faces of sport involvement. SEP emphasizes bodily engagement, self-discipline, and regularized activity. LSS emphasizes co-presence, emotional resonance, and collective experience. Recent evidence suggests that attending live sporting events is associated with higher happiness and lower loneliness (6). Watching sports games has also been linked to subsequent health and happiness in longitudinal evidence from Japan (7). Stadium attendance may further enhance subjective vitality through the experience of sport consumption and team identification (8). However, the key issue is not simply whether sport is beneficial, but how different forms of sport involvement become connected to happiness through daily social life. The more important question is whether these two different pathways both point toward happiness and whether they do so through different social mechanisms.

Despite the growing literature on sport and happiness, several important gaps remain. First, existing research has focused predominantly on active physical participation, while comparatively less attention has been paid to spectator-based sport involvement and its potential relevance to happiness. Second, although LSS involves collective emotion, shared attention, symbolic identification, and interpersonal interaction, the social processes through which these experiences become connected to everyday happiness remain insufficiently understood. Previous research has shown that attendance at sporting events is associated with the size and socioeconomic diversity of social networks, suggesting that spectatorship may generate relational resources beyond the immediate event setting (9). However, existing studies have rarely examined whether these relational resources become embedded in neighborhood social life. Neighborhood social capital (NSC), reflected in everyday interaction, local connectedness, and relational embeddedness, may provide an important bridge in this process. Third, SEP and LSS have seldom been incorporated into the same analytical framework. As a result, it remains unclear whether these two forms of sport involvement are associated with happiness through similar mechanisms or through differentiated pathways. Addressing these gaps is important because it allows sport to be understood not only as an individual health or leisure behavior, but also as a social practice through which bodily activity, collective experience, and everyday social relationships may become interconnected.

Against this background, this study examines the associations of SEP and LSS with happiness and further investigates whether NSC serves as a mediating mechanism linking sport involvement to happiness. Using nationally representative data from CGSS2023, the study places active participation and spectator-based involvement within the same analytical framework and considers both their common association with happiness and their potentially differentiated social pathways. In doing so, this study seeks to make three contributions. First, it extends research on sport and happiness beyond the conventional emphasis on physical participation by incorporating LSS as a distinct form of sport involvement. Second, it introduces NSC as a relational mechanism through which sport-related experiences may become connected to everyday social life and happiness. Third, by distinguishing between the dual-wheel pathway of SEP and the more capital-driven pathway of LSS, the study provides a more socially grounded explanation of how different forms of sport involvement may be associated with happiness. The following section reviews the relevant literature, develops the theoretical framework, and formally proposes the research hypotheses to be tested empirically.

## 2 Literature review and theoretical framework

### 2.1 Sport involvement and happiness: from psychophysical mechanisms to social practice

Existing studies have recognized sport and physical activity as important predictors of happiness and mental health. From a psychophysical perspective, sport is often understood as a bodily tool that improves physical condition, reduces stress, regulates emotions, and enhances happiness. Evidence suggests that physical exercise is associated with better mental health, while recent longitudinal evidence from China also shows that physical activity is positively related to later life satisfaction among middle-aged and older adults (10, 11). These studies establish the basic logic that sport may improve happiness through bodily and psychological mechanisms. Recent studies have further extended this line of research by showing that physical activity and sport-related communication are connected to adolescents’ sport participation attitudes, self-efficacy, social support, and other psychosocial outcomes (12–14).

However, this psychophysical explanation does not fully capture the social nature of sport involvement. Sport does not occur in a social vacuum; it is embedded in specific spaces, routines, interactions, and collective experiences. SEP represents an active form of spatial participation, in which individuals enter gyms, sports venues, or other dedicated exercise spaces and reorganize their bodily routines and everyday order. LSS represents a ritualized form of collective consumption, in which spectators gather around shared symbols and experience synchronized emotions. From the perspective of interaction ritual theory, co-presence, mutual focus, and shared emotional experience can generate emotional energy and social solidarity (15). Therefore, both SEP and LSS should be understood not only as health or leisure behaviors, but also as social practices that may contribute to happiness through different forms of social embeddedness.

### 2.2 The social sedimentation of spatial practice: NSC as a mediating bridge

Modern urban life is often characterized by mobility, segmentation, and weakened neighborhood familiarity. Under such conditions, sport involvement may have a de-atomizing function by creating occasions for individuals to leave private spaces, appear in shared environments, and participate in visible routines. SEP may generate NSC through repeated physical co-presence and the “familiar stranger” effect. People who exercise in the same venues may not immediately become close friends, but repeated encounters can create recognition, weak ties, and low-cost opportunities for interaction. Weak ties are important because they connect individuals beyond intimate circles and expand access to social information and support (16). In this sense, SEP may transform repeated spatial contact into neighborhood familiarity and micro-level trust.

LSS may produce NSC through a different process. Live sport events create temporary communities of spectators who share a team, a symbol, or an event narrative. This shared experience may reduce interpersonal distance and provide common topics for later interaction. The emotional energy generated in the event space can be carried into everyday life and become part of local social interaction. This mechanism is consistent with neighborhood research showing that social capital can mediate the relationship between spatial conditions and residents’ happiness (17). Recent evidence from China also shows that social capital can function as an important mechanism in sport participation, as digital literacy may be linked to rural residents’ sport participation through digital use, human capital accumulation, and social capital (18). Built-environment research also suggests that spatial arrangements influence happiness partly through social capital and everyday interaction (19). Thus, NSC can be understood as the social sedimentation of sport-related spatial practice, transforming temporary encounters and shared experiences into more stable local support and belonging.

### 2.3 Dual-wheel and capital-driven pathways: mechanism differences between SEP and LSS

SEP can be conceptualized as a dual-wheel pathway. On the one hand, it may be associated with happiness through bodily improvement, emotional regulation, self-efficacy, and the reconstruction of everyday order. Structured exercise usually requires regular participation, bodily discipline, and repeated engagement with exercise spaces. These features may help individuals develop a more stable life rhythm and a stronger sense of control. On the other hand, SEP may also be linked to happiness indirectly through social relations generated in sport spaces. Repeated encounters in gyms, sports venues, or community fitness spaces can create opportunities for recognition, interaction, and social support. Recent evidence suggests that physical exercise may reduce loneliness by improving social support and interpersonal relationship quality (20). Sport psychology research also suggests that emotional support, sport confidence, and behavioral regulation are important psychosocial resources within sport settings (21). Therefore, SEP may be associated with happiness through both individual physical-psychological empowerment and the accumulation of NSC.

LSS, by contrast, can be conceptualized as a capital-driven pathway. The emotional excitement of live sports spectatorship is often intense but episodic. Spectators gather in the same space, focus on the same event, and share collective emotions, but the immediate pleasure of cheering, suspense, and excitement may fade after the event ends. From a social psychological perspective, the happiness-related value of LSS is closely connected to shared identity, perceived belonging, and the feeling of being part of a larger collective. Previous research has shown that sport game attendance and team identification can strengthen emotional support, sense of belonging, and subjective well-being, suggesting that spectatorship may generate happiness-related resources through social identification and relational support (22). However, these psychological experiences may remain temporary if they are confined to the event itself. They become more socially meaningful when spectatorship generates continuing communication, interpersonal recognition, and shared topics in everyday life. From a cultural sociological perspective, live sports spectatorship can be understood as an interaction ritual in which co-presence, mutual focus, emotional arousal, symbols, and solidarity are produced among sport fans (23). Yet such emotional energy becomes more relevant to durable happiness only when it is socially sedimented into neighborhood familiarity, micro-level trust, local interaction, and NSC.

Therefore, LSS may rely more heavily on NSC than SEP because its happiness-related value is less rooted in direct bodily practice and more dependent on the social transformation of shared sport experiences. Unlike SEP, which may be linked to happiness through bodily improvement, emotional regulation, self-efficacy, and everyday routine formation, LSS does not necessarily provide direct physical or health-related benefits. Its association with happiness is more likely to emerge when the collective experience of spectatorship is converted into neighborhood interaction, local connectedness, and social belonging. Based on this logic, this study proposes that both SEP and LSS are positively associated with happiness, but through partially different pathways. SEP is expected to show both a direct association with happiness and an indirect association through NSC, whereas LSS is expected to operate mainly through the NSC pathway.

### 2.4 Theoretical framework

**Figure 1:**
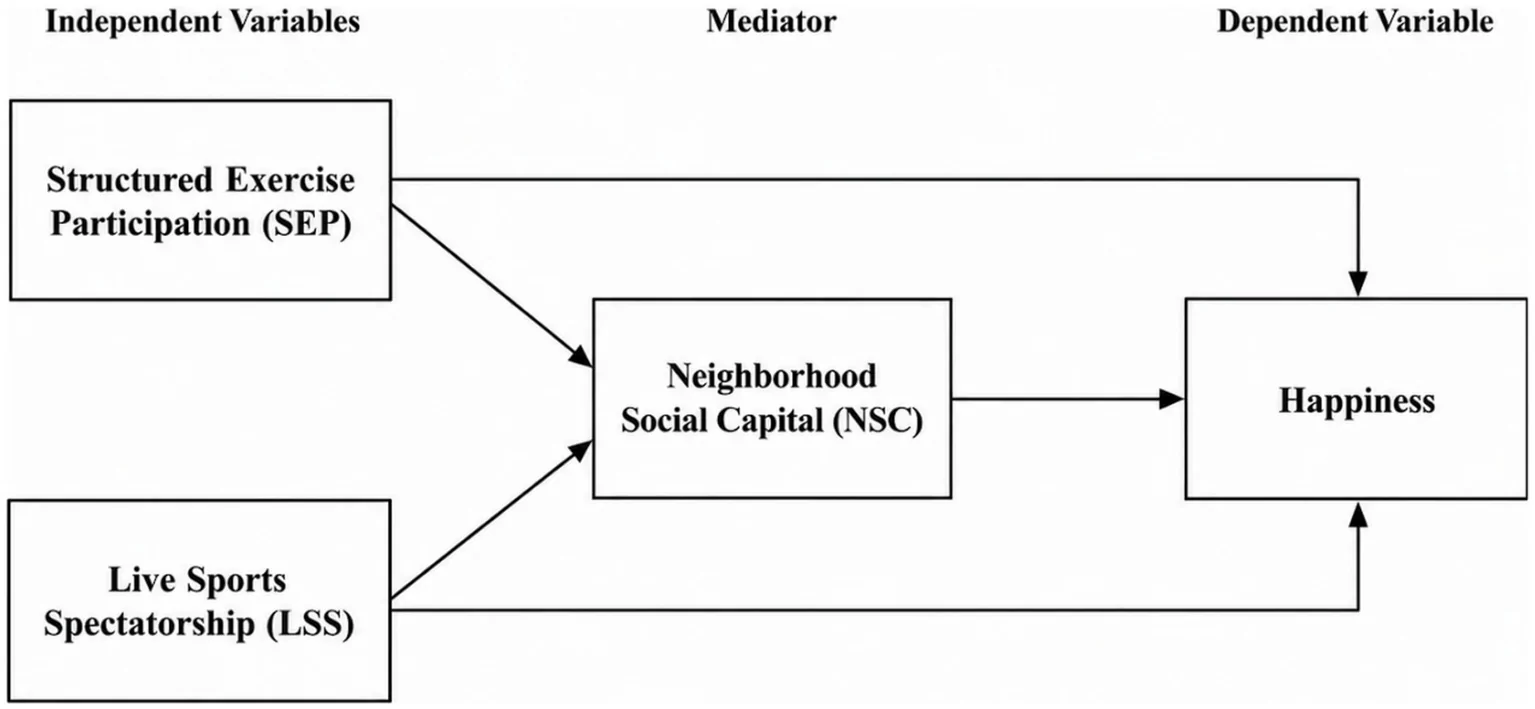
Theoretical framework.

Figure 1 presents the theoretical framework linking sport involvement, NSC, and happiness. The model includes two independent variables, SEP and LSS, one mediator, NSC, and one dependent variable, happiness. SEP and LSS are expected to be positively associated with happiness both directly and indirectly. The indirect pathway operates through NSC, suggesting that sport involvement may enhance happiness by increasing neighborhood-based social interaction, local connectedness, and everyday social support. At the same time, the framework allows for different mechanisms between the two forms of sport involvement: SEP may contribute to happiness through both individual physical-psychological benefits and social capital accumulation, whereas LSS may rely more strongly on the transformation of shared sport experiences into neighborhood social capital. Thus, the framework highlights sport as not only a health or leisure behavior, but also a social practice embedded in everyday relationships. Based on this theoretical framework, the following hypotheses are proposed:

*H1*: SEP is positively associated with happiness.

*H2*: LSS is positively associated with happiness.

*H3*: NSC mediates the association between SEP and happiness.

*H4*: NSC mediates the association between LSS and happiness.

## 3 Materials and methods

### 3.1 Data resource

This study uses data from the 2023 Chinese General Social Survey (CGSS2023), a nationally representative survey that covers a wide range of topics, including demographic characteristics, family social conditions, health, social interaction, leisure activities, and happiness. The original CGSS2023 sample contains 11,326 respondents. However, the smaller analytical sample in this study should not be interpreted as indicating that nearly half of the national survey data were invalid. Rather, CGSS2023 contains different questionnaire modules, and the leisure activity module relevant to this study was administered to 6,987 respondents. Therefore, the reduction in sample size mainly reflects the modular design of CGSS2023 rather than a general data quality problem.

On this basis, we further excluded cases with missing, invalid, “refused to answer,” or “do not know” responses on the key variables, including structured exercise participation, live sports spectatorship, happiness, and self-rated health. After this step, the sample was reduced to 5,887 observations. We then restricted the data to respondents with valid information on the control variables and the neighborhood interaction measure. The final analytical sample consisted of 5,451 respondents. Thus, the final sample should be understood as the complete-case analytical sample for the variables used in this study.

### 3.2 Dependent variable

Happiness was measured using a single-item self-reported evaluation of respondents’ overall life state. Respondents were asked, “Overall, do you think your life is happy?” Responses were coded on a five-point scale ranging from 1 = “very unhappy” to 5 = “very happy,” with higher values indicating higher levels of happiness. This measure does not capture clinical mental health symptoms or a multidimensional psychological construct. Rather, it reflects respondents’ general subjective evaluation of their current life condition. Single-item measures of happiness and life satisfaction are widely used in large-scale social surveys because they provide a concise and comparable indicator of overall happiness, especially when the research focus is on population-level associations rather than psychological diagnosis (24–26). Responses coded as “do not know” or “refused to answer” were treated as missing and excluded from the analysis. In the main analysis, happiness was treated as a quasi-continuous outcome and estimated using OLS regression. As a robustness check, the variable was also modeled as an ordinal outcome using ordered logistic regression.

### 3.3 Independent variables

This study distinguishes between two forms of sport involvement: SEP and LSS. SEP captures respondents’ active engagement in exercise in dedicated sports venues or gyms. It was measured by asking respondents whether the statement “I often go to dedicated sports venues or gyms to exercise” fit their actual lifestyle or true beliefs. Responses were recorded on a four-point scale ranging from “strongly agree” to “strongly disagree.” To facilitate interpretation, this variable was reverse coded so that higher values indicate greater participation in structured exercise. LSS captures respondents’ involvement in sport as spectators. It was measured by asking how often respondents attended live sports events during their leisure time in the past year. Responses were originally coded on a five-point scale from “every day” to “never.” This variable was also reverse coded so that higher values indicate more frequent live sports spectatorship. Taken together, SEP and LSS reflect two distinct modes of sport involvement, namely active participation and passive spectatorship.

### 3.4 Mediator variables

The mediator is NSC, operationalized through the frequency of neighborhood social interaction. Respondents were asked how often they engaged in social and leisure activities with their neighbors, such as visiting one another, watching television together, having meals together, or playing cards. Responses were originally coded on a seven-point scale from 1 = “almost every day” to 7 = “never.” The variable was reverse coded so that higher values indicate more frequent neighborhood interaction. In this sense, NSC in the present study reflects respondents’ everyday social connectedness and interactional embeddedness within the neighborhood.

### 3.5 Control variables

A range of demographic, socioeconomic, and health-related variables were included as controls. These variables comprise gender (male = 1), age (in years), education (coded into five categories from no formal education to college and above), marital status (married = 1), political status (mass affiliation = 1), ethnicity (Han = 1), self-rated health, family economic status, and type of residential area. Residential area was measured as a four-category variable distinguishing urban center, urban fringe or rural–urban fringe, town, and rural area. These covariates were included to account for individual differences in social position, living context, and health conditions that may be associated with both sport involvement and happiness.

### 3.6 Analytical strategy

The analysis proceeded in four steps. All statistical analyses were conducted using Stata 17.0. First, descriptive statistics and frequency analyses were used to summarize the main variables and the characteristics of the analytical sample. Continuous and ordinal variables were reported using means, standard deviations, minimum values, and maximum values, while categorical variables were reported using frequencies and percentages.

Second, OLS regression models were estimated as the main analytical approach to test H1 and H2, namely whether SEP and LSS were positively associated with happiness. Happiness was treated as a quasi-continuous outcome in the main analysis. The models were estimated sequentially by first including the control variables only, then adding SEP and LSS separately, and finally including both variables simultaneously. Robust standard errors were used in all OLS models to account for potential heteroskedasticity.

Third, because happiness was measured on a five-category ordered scale, ordered logistic regression models were estimated as a robustness check for H1 and H2. This step was used to assess whether the associations between sport involvement and happiness remained substantively unchanged when the ordinal nature of the dependent variable was taken into account.

Finally, bootstrap mediation analysis was conducted to test H3 and H4, namely whether NSC mediated the association between SEP and happiness and the association between LSS and happiness. Specifically, bootstrap procedures were used to estimate the indirect, direct, and total effects of SEP and LSS on happiness through NSC. Bias-corrected confidence intervals were used to assess the statistical significance of the mediation effects.

## 4 Result

### 4.1 Descriptive statistics

Table 1 and Table 2 present the descriptive statistics of the analytical sample. The final sample included 5,451 respondents. Table 1 reports the descriptive statistics for the main variables and other continuous or ordinal variables. Overall, respondents reported a relatively high level of happiness, with a mean value of 3.90. By contrast, both forms of sport involvement appeared to be less common in everyday life. The mean value of SEP was 1.48, suggesting that regular gym-based or venue-based exercise was not a widely established routine among respondents. The mean value of LSS was 1.29, indicating that attending live sports events was also a relatively uncommon leisure activity. NSC had a mean value of 3.86 and a standard deviation of 2.25, showing substantial variation in respondents’ neighborhood-based social interaction. The average age of respondents was 54.50 years, indicating a relatively middle-aged and older sample structure. Respondents reported a moderate level of self-rated health, with a mean value of 3.35, and a family economic status mean of 2.56. The average education level was 3.08.

**Table 1:** Continuous variables.

Variable	N	Mean	SD	Min	Max
Happiness	5,451	3.90	0.85	1	5
SEP	5,451	1.48	0.76	1	4
LSS	5,451	1.29	0.66	1	5
NSC	5,451	3.86	2.25	1	7
Age	5,451	54.50	16.32	18	94
Self-rated health	5,451	3.35	1.14	1	5
Family economic status	5,451	2.56	0.74	1	5
Education	5,451	3.08	1.25	1	5

**Table 2:** Categorical variables.

Variable	Category	*N*	%
Gender	Female	3,008	55.16
	Male	2,443	44.84
Marital status	Not married	1,557	28.56
	Married	3,894	71.44
Political status	Other	867	15.91
	Mass affiliation	4,584	84.09
Ethnicity	Minority	507	9.3
	Han	4,944	90.7
Residential area	Urban center	1,368	25.1
	Urban fringe or rural–urban fringe	913	16.75
	Town	558	10.24
	Rural	2,612	47.92

Table 2 reports the frequency distributions of categorical demographic and residential variables. Among the 5,451 respondents, 3,008 were female, accounting for 55.16% of the sample, while 2,443 were male, accounting for 44.84%. A total of 3,894 respondents were married, accounting for 71.44%, while 1,557 respondents were not married, accounting for 28.56%. Regarding political status, 4,584 respondents reported mass affiliation, accounting for 84.09% of the sample. In terms of ethnicity, 4,944 respondents were Han, accounting for 90.70%. The residential distribution showed that 1,368 respondents lived in urban centers, 913 in urban fringe or rural–urban fringe areas, 558 in towns, and 2,612 in rural areas, accounting for 25.10, 16.75, 10.24, and 47.92% of the sample, respectively. Taken together, the descriptive results suggest that the analytical sample was characterized by relatively high happiness, relatively limited sport involvement, a relatively older age structure, and considerable heterogeneity in neighborhood-based social interaction.

### 4.2 Main regression results

Table 3 reports the OLS regression results for the association between sport involvement and happiness. Model 1 includes only the control variables, Models 2 and 3 add SEP and LSS separately, and Model 4 includes both variables simultaneously. These models were used to test H1 and H2. H1 proposed that SEP is positively associated with happiness, while H2 proposed that LSS is positively associated with happiness.

**Table 3:** Effects of gym-based exercise and live sports spectatorship on happiness.

Variables	Model 1	Model 2	Model 3	Model 4
SEP		0.061*** (0.014)		0.054*** (0.014)
LSS			0.047** (0.016)	0.034* (0.016)
Gender	−0.052** (0.022)	−0.059*** (0.022)	−0.057** (0.022)	−0.061*** (0.022)
Age	0.006*** (0.001)	0.006*** (0.001)	0.006*** (0.001)	0.006*** (0.001)
Education	0.048*** (0.012)	0.045*** (0.012)	0.045*** (0.012)	0.043*** (0.012)
Marital status	0.079*** (0.025)	0.081*** (0.025)	0.080*** (0.025)	0.082*** (0.025)
Political status	−0.108*** (0.027)	−0.104*** (0.027)	−0.103*** (0.027)	−0.101*** (0.027)
Ethnicity	−0.002 (0.040)	0.002 (0.039)	0.002 (0.040)	0.004 (0.039)
Self-rated health	0.150*** (0.011)	0.147*** (0.011)	0.149*** (0.011)	0.147*** (0.011)
Family economic status	0.232*** (0.018)	0.227*** (0.018)	0.230*** (0.018)	0.227*** (0.018)
Urban fringe	0.107*** (0.032)	0.116*** (0.032)	0.110*** (0.032)	0.117*** (0.032)
Residential area				
Town	0.124*** (0.036)	0.133*** (0.037)	0.121*** (0.037)	0.130*** (0.037)
Rural	0.089*** (0.029)	0.109*** (0.029)	0.094*** (0.029)	0.110*** (0.029)
Constant	2.326*** (0.108)	2.225*** (0.111)	2.262*** (0.111)	2.190*** (0.113)
*N*	5,451	5,451	5,451	5,451
*R* ^2^	0.115	0.1176	0.1163	0.1182

* *p* < 0.05, ** *p* < 0.01, *** *p* < 0.001.

The results provide empirical support for both hypotheses. In Model 2, SEP has a positive and statistically significant coefficient (*β* = 0.061, *p* < 0.001), indicating that respondents with higher levels of SEP reported higher levels of happiness after adjusting for demographic, socioeconomic, health-related, and residential characteristics. This result supports H1. In Model 3, LSS is also positively associated with happiness (*β* = 0.047, *p* < 0.01), suggesting that more frequent attendance at live sports events is related to higher happiness. This result supports H2. When SEP and LSS are included simultaneously in Model 4, both coefficients remain positive and statistically significant, with SEP at 0.054 (*p* < 0.001) and LSS at 0.034 (*p* < 0.05). These results indicate that SEP and LSS each have an independent positive association with happiness, even after adjusting for the other form of sport involvement. Therefore, the OLS models provide consistent support for H1 and H2.

The control variables also show meaningful patterns. Age, education, marital status, self-rated health, and family economic status are positively associated with happiness across the models. Respondents living in urban fringe areas, towns, and rural areas report higher happiness than those living in urban centers. By contrast, gender and political status show negative associations with happiness, while ethnicity is not statistically significant. Overall, the OLS results provide initial evidence that both active and spectator-based sport involvement are positively related to happiness after controlling for a broad set of individual and contextual characteristics Table 3 about here.

### 4.3 Robustness check using ordered logistic regression

Table 4 presents the ordered logistic regression results. Because happiness is an ordered outcome, ordered logistic models were estimated to examine whether the main findings remain stable under an alternative model specification. Models 5 and 6 include SEP and LSS separately, while Model 7 includes both variables simultaneously. The results are highly consistent with the OLS estimates. SEP is positively and significantly associated with happiness in Model 5 (*β* = 0.143, *p* < 0.001), and LSS also shows a positive and significant association with happiness in Model 6 (*β* = 0.139, *p* < 0.001). These findings indicate that both forms of sport involvement are associated with a higher likelihood of reporting greater happiness.

**Table 4:** Ordered logistic regression results for happiness.

Variables	Model 5	Model 6	Model 7
SEP	0.143*** (0.037)		0.121** (0.038)
LSS		0.139** (0.040)	0.109** (0.041)
Control variables	YES	YES	YES
cut1	−0.190 (0.286)	−0.237 (0.284)	−0.073 (0.289)
cut2	1.294*** (0.273)	1.246*** (0.271)	1.411*** (0.277)
cut3	2.784*** (0.272)	2.734*** (0.270)	2.901*** (0.276)
cut4	5.551*** (0.283)	5.500*** (0.281)	5.671*** (0.287)
Pseudo R^2^	0.048	0.047	0.048

*p < 0.05, ** *p* < 0.01, *** *p* < 0.001.

When SEP and LSS are included together in Model 7, both variables remain statistically significant. The coefficient of SEP is 0.121 (*p* < 0.01), and the coefficient of LSS is 0.109 (*p* < 0.01). This suggests that the positive associations between sport involvement and happiness are not sensitive to the choice between OLS and ordered logistic regression. Overall, the robustness check provides further support for the main finding that both active and spectator-based forms of sport involvement are positively associated with happiness among Chinese respondents.

### 4.4 Mediation analysis

Table 5 presents the bootstrap mediation results for the role of NSC. These models were used to test H3 and H4. H3 proposed that NSC mediates the association between SEP and happiness, while H4 proposed that NSC mediates the association between LSS and happiness.

**Table 5:** Bootstrap mediation analysis of neighborhood interaction.

Effect	Coefficient	95% CI (BC)
Panel A. SEP → NSC → Happiness		
Indirect effect	0.0016	[0.0004, 0.0040]
Direct effect	0.0524	[0.0222, 0.0781]
Total effect	0.0539	[0.0242, 0.0807]
Panel B. LSS → NSC → Happiness		
Indirect effect	0.0025	[0.0006, 0.0057]
Direct effect	0.0311	[−0.0011, 0.0639]
Total effect	0.0335	[0.0022, 0.0655]

For SEP, the indirect effect through NSC was positive and statistically significant, as the bias-corrected 95% confidence interval did not include zero [*β* = 0.0016, 95% CI (0.0004, 0.0040)]. This suggests that SEP was associated with happiness partly through neighborhood-based social capital. At the same time, the direct effect of SEP remained positive and significant [*β* = 0.0524, 95% CI (0.0222, 0.0781)], and the total effect was also significant [*β* = 0.0539, 95% CI (0.0242, 0.0807)]. These results support H3. This pattern is also consistent with the proposed dual-wheel pathway: SEP is related to happiness not only through its direct physical and psychological implications, but also through the social connections generated in shared exercise spaces.

For LSS, the indirect effect through NSC was also positive and significant [*β* = 0.0025, 95% CI (0.0006, 0.0057)]. This indicates that LSS was associated with happiness through the accumulation of neighborhood-based social capital. However, after NSC was included in the mediation model, the direct effect of LSS was not statistically significant, as the confidence interval included zero [*β* = 0.0311, 95% CI (−0.0011, 0.0639)]. The total effect remained positive and significant [*β* = 0.0335, 95% CI (0.0022, 0.0655)]. These results support H4. This pattern is consistent with the idea of a capital-driven pathway, in which the happiness-related value of LSS depends largely on whether shared sport experiences are translated into neighborhood interaction, local connectedness, and micro-level trust.

Overall, the mediation results suggest that NSC may serve as an important explanatory mechanism linking sport involvement to happiness. SEP and LSS are both connected to happiness through NSC, but their mediation patterns differ. SEP shows both a direct pathway and an indirect pathway through NSC, whereas LSS appears to operate mainly through the NSC pathway. These findings provide empirical support for H3 and H4, and further support the central argument of this study that sport involvement is linked to happiness not only as a form of physical activity or leisure consumption, but also as a social practice embedded in everyday neighborhood relationships.

## 5 Discussion

### 5.1 Associations between sport involvement and happiness

The first major finding of this study concerns the associations between sport involvement and happiness. The OLS results showed that both SEP and LSS were positively associated with happiness after controlling for demographic, socioeconomic, health-related, and residential characteristics, providing empirical support for H1 and H2. The ordered logistic regression results further confirmed that these associations remained stable when happiness was treated as an ordinal outcome. Therefore, the positive relationship between sport involvement and happiness is not limited to a specific model specification, but appears consistently across both the main regression model and the robustness check. The finding that SEP is positively associated with happiness is consistent with previous evidence that sport participation is related to higher subjective well-being (27). More recent studies have also shown that physical activity is associated with higher happiness and better mental health outcomes (28). In the Chinese context, sport participation has been found to be associated with happiness through health and social status-related mechanisms (29). Physical activity may also be linked to mental health through multiple psychosocial pathways, including emotional regulation, self-efficacy, social support, and social connection (30). In this study, SEP reflects a structured form of exercise embedded in gyms or dedicated sport venues. Such participation is not only a matter of bodily movement, but also a routinized everyday practice that may help individuals organize time, maintain a sense of control, and access shared activity spaces. In this sense, the positive association between SEP and happiness suggests that structured exercise may be linked to happiness through both physical-psychological resources and everyday life order.

The positive association between LSS and happiness further broadens the meaning of sport involvement. Unlike SEP, LSS does not involve direct physical exercise, but it provides a different form of sport-related experience through co-presence, shared attention, collective emotion, and symbolic identification. Watching or attending sports events may be associated with higher happiness through emotional arousal, social interaction, and collective enjoyment (31). Evidence from a multi-method study also suggests that watching sport is related to happiness through positive emotional and reward-related experiences (32). Studies on live sport attendance further show that spectatorship can be associated with happiness, vitality, and reduced loneliness (33). From a social psychological perspective, identification with sport teams or groups may provide belonging, shared meaning, and social psychological benefits (34). Therefore, LSS should not be understood simply as passive entertainment, but as a socially organized leisure practice. This finding supports H2 and suggests that the happiness-related value of sport is not limited to active bodily participation. Sport involvement may also be linked to happiness through shared spaces, collective experiences, and socially embedded leisure. Taken together, the results related to H1 and H2 indicate that both active and spectator-based forms of sport involvement are positively associated with happiness. This finding supports the broader argument of this study that sport should be understood not only as physical activity or leisure consumption, but also as a social practice that connects bodily experience, emotional experience, and everyday social life.

### 5.2 Mediating role of NSC and differentiated pathways

The second major finding concerns the mediating role of NSC. The bootstrap mediation results showed that NSC mediated the association between SEP and happiness, providing empirical support for H3. NSC also mediated the association between LSS and happiness, supporting H4. These findings suggest that neighborhood-based social interaction may be an important pathway through which sport involvement is linked to happiness. This interpretation is consistent with social capital theory, which emphasizes that social networks, trust, reciprocity, and everyday interaction can provide resources related to individual happiness (35). Earlier research has also shown that the social context of happiness is shaped by the quality of interpersonal ties and local embeddedness (36). In the Chinese context, community social capital has been found to be associated with happiness through psychological flourishing and everyday social support (37). Evidence based on CGSS data also suggests that social trust and social capital are closely related to residents’ happiness (38). In this study, NSC is operationalized through the frequency of neighborhood social interaction. Although this measure does not capture all dimensions of social capital, such as trust, reciprocity, or perceived support, it reflects an important behavioral dimension of everyday connectedness. The significant indirect effects therefore suggest that sport involvement may be associated with happiness partly through stronger neighborhood-based social embeddedness.

The mediation patterns further indicate that SEP and LSS are connected to happiness through different social pathways. For SEP, the indirect effect through NSC was significant, while the direct effect remained significant. This pattern is consistent with the proposed dual-wheel pathway, suggesting that SEP is associated with happiness both directly and indirectly through neighborhood-based social connections. Structured exercise may be linked to happiness through physical health, emotional regulation, self-efficacy, and the reconstruction of daily routines, while also creating repeated encounters and informal interaction in shared exercise spaces. Sport engagement has been shown to be associated with the formation of social capital by expanding social networks and increasing opportunities for interaction (39). Recent work also highlights the role of social capital in linking sport and physical activity contexts to happiness (40). For LSS, the indirect effect through NSC was significant, whereas the direct effect was not statistically significant after NSC was included. This pattern is consistent with the proposed capital-driven pathway, suggesting that LSS may be linked to happiness mainly through the transformation of shared sport experiences into social connection, common topics, neighborhood interaction, and micro-level trust. Sport spectatorship can be associated with social networks and social trust, especially when spectatorship becomes part of collective leisure and shared identity (41). Watching or attending sports events may also be linked to happiness through emotional experience and social interaction. Taken together, the findings related to H3 and H4 highlight the social nature of sport involvement: sport is linked to happiness not only as physical activity or entertainment, but also as a social practice that connects individuals to shared spaces, collective experiences, and everyday neighborhood relationships.

### 5.3 Practical implications

The findings have several practical implications. Efforts to promote happiness through sport should move beyond simply encouraging individuals to exercise more frequently and should instead pay greater attention to the social environments in which sport activities occur. Community fitness spaces, accessible sports venues, and inclusive exercise programs can help residents develop regular sport routines while also creating opportunities for repeated interaction and neighborhood-based connection. The finding on LSS further suggests that live sports events should not be viewed only as commercial entertainment, but also as potential public resources for shared leisure, collective emotion, and social participation. Small-scale, affordable, and community-based sport events may be especially useful in communities where everyday interaction is weak. More importantly, the mediating role of NSC indicates that sport-based interventions should be designed not only to improve physical activity or leisure participation, but also to foster communication, familiarity, and local trust. In this sense, sport can serve as both a health-promotion tool and a community-building strategy, helping strengthen social ties and improve everyday happiness (37).

### 5.4 Limitations and future research

Several limitations should be acknowledged. First, this study uses cross-sectional data, so the results should be interpreted as associations rather than causal effects. Although SEP, LSS, NSC, and happiness are significantly related, the direction of these relationships cannot be definitively established. Reverse causality is possible, as happier individuals may be more willing to participate in structured exercise, attend live sports events, or interact with neighbors. Therefore, causal wording should be used with caution when interpreting the findings. Future research could use longitudinal data, panel designs, or quasi-experimental approaches to better examine the causal direction between sport involvement, NSC, and happiness.

Second, happiness is measured by a single self-reported item. This measure is suitable for capturing respondents’ general evaluation of their life state in a large-scale social survey, but it does not represent a clinical or multidimensional psychological assessment. Future research could use multidimensional measures of subjective well-being, mental health, or psychological functioning to further examine how sport involvement is related to different aspects of well-being.

Third, the measurements of SEP and LSS are relatively general and are not directly comparable. SEP was measured by a four-point agreement item concerning gym-based or venue-based exercise, whereas LSS was measured by the frequency of attending live sports events during the past year. Because these two variables differ in both scale structure and substantive meaning, their coefficients should not be directly compared as effect sizes. The results should instead be interpreted as evidence that each form of sport involvement is associated with happiness within its own measurement framework. Future studies could adopt more comparable indicators, such as frequency-based, duration-based, or time-use measures for both active sport participation and sport spectatorship. More detailed measures of exercise intensity, sport type, event type, participation quality, and spectatorship context would also help clarify the differences between active and spectator-based sport involvement.

Fourth, NSC is operationalized through the frequency of neighborhood social interaction. Although this captures an important behavioral dimension of neighborhood social capital, it does not fully measure other key dimensions, such as trust, reciprocity, perceived support, or community belonging. As a result, the mediation results should be interpreted as evidence for a neighborhood interaction pathway rather than as a complete test of the multidimensional concept of social capital. This measurement limitation may lead to a partial representation of the broader social capital mechanism. Future research could use multidimensional measures of neighborhood social capital to further clarify how different forms of local relationships mediate the link between sport involvement and happiness.

## 6 Conclusion

Using data from CGSS2023, this study examined the relationships among SEP, LSS, NSC, and happiness. The findings show that both SEP and LSS are positively associated with happiness, suggesting that sport involvement matters not only through active physical exercise but also through spectator-based leisure experiences. The mediation analysis further indicates that NSC plays an important role in linking sport involvement to happiness. SEP is associated with happiness through both a direct pathway and an indirect pathway via NSC, while LSS appears to operate mainly through the NSC pathway. These findings highlight the social meaning of sport in everyday life. Sport is not merely a health behavior or a form of leisure consumption; it is also a social practice that connects individuals to shared spaces, collective experiences, and neighborhood-based relationships. By showing how different forms of sport involvement are embedded in social interaction and local connectedness, this study provides evidence that sport can contribute to happiness through both individual and relational pathways.

## Data Availability

Publicly available datasets were analyzed in this study. This data can be found here: The data used in this study are from the 2023 wave of the Chinese General Social Survey (CGSS), which is publicly available at the CGSS data repository (http://cgss.ruc.edu.cn/). Researchers can access the dataset by registering on the official website.
